# Prone Cardiopulmonary Resuscitation (CPR) Protocol: A Single-Center Experience at Implementation and Review of Literature

**DOI:** 10.7759/cureus.29604

**Published:** 2022-09-26

**Authors:** Cameron McCraw, Caroline Baber, Aaron H Williamson, Yanwei Zhang, Rachel S Sinit, Ann D Alway, Shikha Jain, Nitesh K Jain, Kovid Trivedi

**Affiliations:** 1 Pulmonology and Critical Care, Western University of Health Sciences, Lebanon, USA; 2 Critical Care, Salem Health, Salem, USA; 3 Internal Medicine, M.V.J. Medical College and Research Hospital, Bengaluru, IND; 4 Critical Care Medicine, Mayo Clinic Health System, Mankato, USA; 5 Pulmonary/Critical Care Medicine, Salem Pulmonary Associates/Salem Health, Salem, USA

**Keywords:** quality improvement and patient safety, resuscitation education, cardiac resuscitation, cardiac arrest, in hospital cardiac arrest, prone cpr, prone position, cardiopulmonary resuscitation

## Abstract

The prone position is a crucial position used in the operating rooms and the intensive care units, with its importance highly recognized during the COVID-19 pandemic in patients with acute respiratory distress syndrome (ARDS). Cardiopulmonary resuscitation (CPR) is a cardinal procedure that is indicated and performed on any eligible patient who has cardiopulmonary arrest and resultant lack of perfusion and oxygenation. When a patient has a cardiopulmonary arrest in the prone position, the options include rotating the patient supine before starting cardiopulmonary resuscitation (CPR) or beginning CPR while prone. Prone CPR has not had a widely accepted use so far. In this article, we narrate the process of protocol development and staff education at our hospital for the initiation of prone CPR and review the literature related to it. Prone CPR is an effective technique with good outcomes and involves a learning curve. Appropriate training needs to be done before implementing the protocol, and adequate quality control measures need to be set to ensure that the skill set is maintained.

## Introduction and background

Prone position has been a critical position for multiple surgeries in the operating room (OR) as well as in the management of acute respiratory distress syndrome (ARDS) management [1] in intensive care units (ICUs). With the COVID-19 pandemic, the importance of prone positioning of patients has increased as the number of patients in ARDS has increased substantially. These patients are often sicker than their cohorts with other ICU illnesses and at a high risk of cardiopulmonary arrest. Old-time wisdom had recommended supine-positioning the patient before initiating cardiopulmonary resuscitation (CPR). However, it is a time and labor-intensive process. During cardiopulmonary arrest, CPR must be performed on the patient promptly to assist with adequate circulation and perfusion of the vital organs. Delayed initiation of CPR leads to worse outcomes. Prone CPR has had increasing acceptance over the last years and is also now endorsed by the American Heart Association/American College of Cardiology (AHA/ACC) Advanced Cardiac Life Support (ACLS) 2020 updated guidelines [2]. We describe the methodology used to implement the protocol of prone CPR in the ICU at our hospital, the challenges encountered during the process, and review related literature. 

## Review

Materials and methods

With an increasing number of critically ill patients with ARDS placed pronely, there was a need to develop a protocol to facilitate the performance of prone CPR. Salem Hospital is a 494-bed acute care hospital located in Salem, Oregon, United States of America (USA), with the highest number of COVID-19 patients admitted in the state during the COVID-19 pandemic. During the public health emergency, the bed capacity was increased to accommodate the increased patient load. The hospital has 42 ICU beds with the capability to expand ICU bed capacity, which was done during the pandemic’s peak. Code Blue Committee is the designated workgroup at the hospital that oversees policy-making, training, compliance, equipment-related issues, and quality control about the CPR-related process across the hospital system. The nursing and physician leaders of the code-blue committee, along with an ethicist, reviewed the literature on prone CPR and defined the data into five categories based on the 2017 Johns Hopkins Hospital/Johns Hopkins University School of Nursing Evidence Level and Quality Guide [3]. 

Based on the available literature citing the effectiveness of prone CPR and the negative impacts of time delay and personnel involved in turning the patient supine, the data supported the development of a protocol for prone CPR. Data was presented to the intensivists (12) along with the intensive care unit (ICU), rapid response team (RRT), and respiratory therapy (RT) leadership.

Early palliative care consult was highly encouraged as soon as the decision was made to prone position a patient with invasive mechanical ventilation.

An appropriate patient was defined as a “full code” patient that is physically in the medical ICU or cardiovascular ICU who has a cardiopulmonary arrest while in the prone position with a secure advanced airway (endotracheal tube or tracheostomy tube) and still requires mechanical ventilation. The patient should not have a contraindication to prone CPR (e.g., spinal injury, active bleeding, absence of adequately trained staff, etc.). The code team leader was given the authority to decide on continuing prone CPR or switching to the supine position during the resuscitation process.

ICU personnel, including physicians, physician assistants, bedside nurses, rapid response nurses, respiratory therapists, and the “lift team” (that helps with chest compressions and patient positioning), were trained in the process of prone CPR. A 1000 ml saline bag was kept at the bedside of prone patients at all times to be placed under the sternum before initiation of the compressions.

A limited volume of patients requiring prone CPR was one of the biggest challenges in keeping up the skills related to the process. To help overcome this, the process of prone CPR was reiterated by the charge nurse with the bedside nurse of all prone patients at the start of every shift. In addition, at the beginning of each shift, potential patients were reviewed by the physician-led interdisciplinary team during rounds. A pictorial of the hand and defibrillation pad placement was placed on each code cart and reviewed with the bedside nurse.

Technique

Supine CPR: Formal guidelines have been developed to document standard precordial cardiac compression procedures for patients with cardiac arrest in a supine position. During compressions, the rescuer's hands are placed over an external chest location to generate maximal blood flow ejected from the left ventricle into the aorta. For instance, the lower half of the sternum of an adult victim's chest has been identified to be the optimal site for compression on victims in a supine position. To deliver a high-quality supine CPR, a few critical components have been recognized, including 1) interruptions between compressions should be minimized, and 2) compressions of adequate depth and rate should be provided to generate maximal and continuous blood flow into the aorta from the patient's left ventricle.

Challenges with repositioning to supine: Time spent rotating a patient of cardiopulmonary arrest is time not spent delivering life-saving compressions. Rotating a patient may pose challenges: patients may be large or otherwise heavy, requiring multiple rescuers to assist, or a patient may already have attached medical devices that must remain connected (rotating thus must be coordinated carefully). Any communicable diseases the patient may have, such as COVID-19, further increases the risk of exposure to involved rescuers. Lastly, in some circumstances, the patient cannot be rotated at all. Some surgical procedures require fixation of the skull or other body parts while the patient is in a prone position due to the procedure.

**Figure 1:**
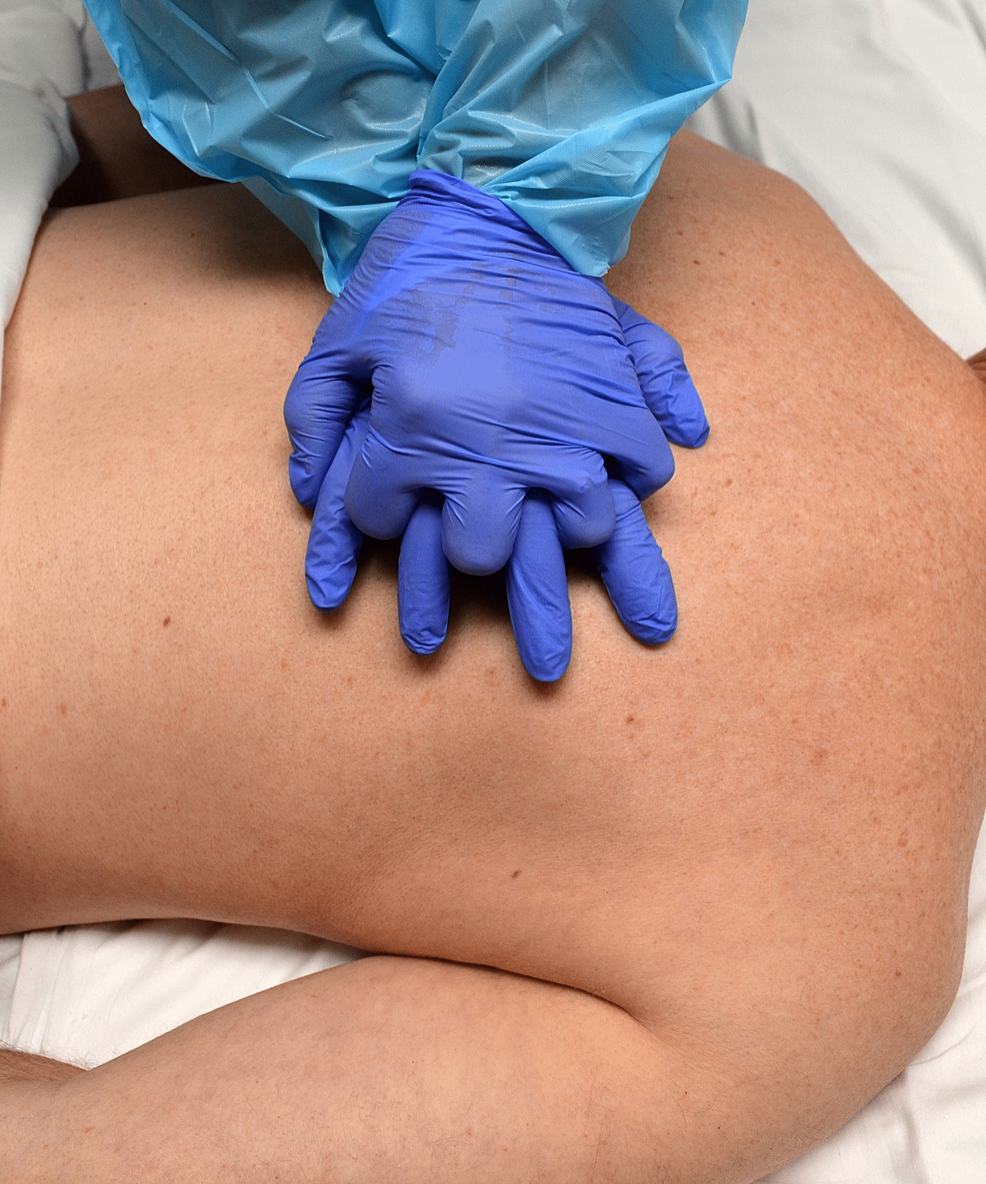
Hand Position During Prone CPR: Over the T7-T10 Vertebral Bodies or 0-2 Vertebral Segments Below the Inferior Angle of the Scapula Pictures were taken by the marketing department of Salem hospital on a volunteer to show the position of hand placement.

Prone CPR:** **The principle guiding high-quality prone CPR remains the same as that for supine CPR: generating maximal and continuous blood flow into the aorta. The rate and depth of compression remain the same as supine CPR. Continuous feedback measures like end-tidal carbon dioxide (ET-CO2) can be used to define effective compressions. Anatomical landmarks have been identified for optimal hand placement to generate high-quality CPR compressions for patients in a prone position: "the level where the cardiac cross-section is largest is 0 to 2 vertebral segments below the inferior angle of the scapula", effectively, over the T7 through T10 vertebral bodies (Figure 1) [2,4]. 

Prone CPR’s effectiveness may increase if the anterior abdominal wall remains in contact with a firm surface during compressions as it restricts movement of the abdominal structures. This is thought to augment the force delivered to the ventricles and the rapid reduction of intrathoracic volume. Therefore, placing a firm object under the sternum is recommended to provide effective counterforce during compression. A sandbag or a bag of saline is sufficient for this purpose [5]. The effectiveness of CPR is affected by the depth of chest compressions, and therefore it is still recommended to place the patient on a hard surface or to place a rigid board under the patient [6].

**Figure 2:**
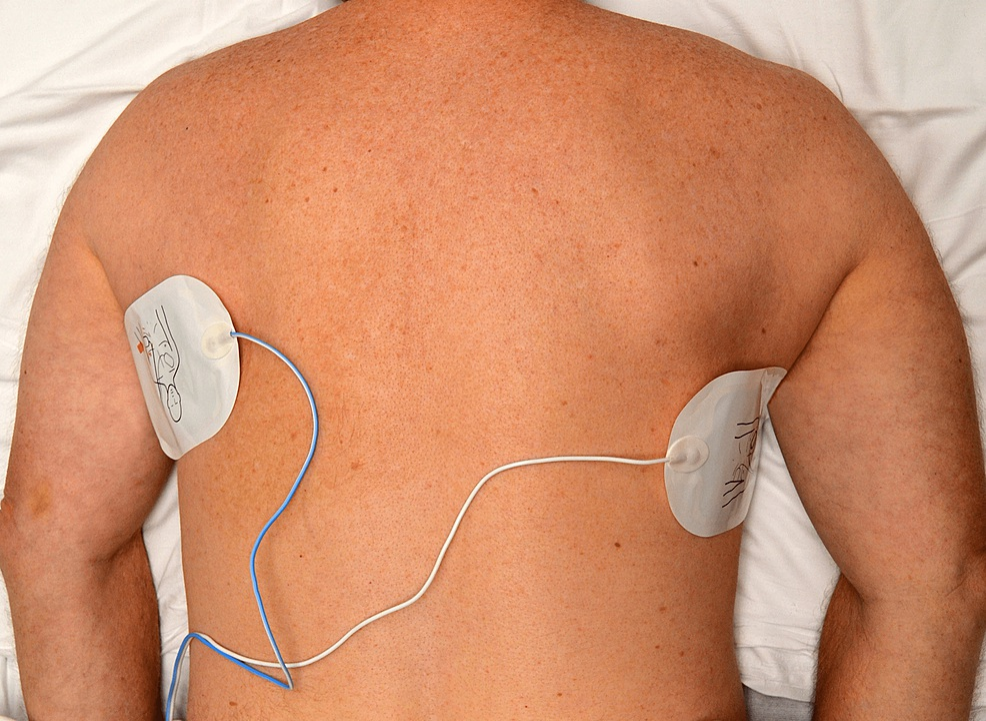
Defibrillator Pad Placement in the Side-to-Side Position: Under Both Armpits Pictures were taken by the marketing department of Salem hospital on a volunteer to show the position of defibrillator pad placement.

**Figure 3:**
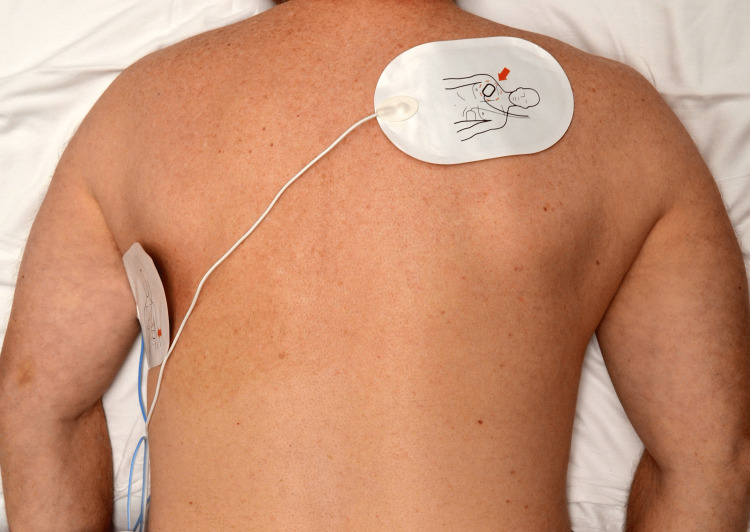
Defibrillator Pad Placement in Left Shoulder-Right Axilla Position Pictures were taken by the marketing department of Salem hospital on a volunteer to show the position of the defibrillator pad placement.

To facilitate the conduction of electrical current effectively through the heart, defibrillation pad placement in the prone position follows the same principles as used during conventional supine CPR. The pads can be placed on the patient’s two sides under the armpits (Figure 2) or the left shoulder and right axilla positions (Figure 3) [5].

Discussion

Studies have shown that prone positioning increases ventilation and can benefit patients with severe acute respiratory distress syndrome (ARDS) by increasing oxygen saturation and reducing mortality [7]. A meta-analysis by Mir et al. showed that 621 out of 2891 hospitalized COVID-19 patients underwent a cardiac arrest (pooled prevalence 8%), and only 52 survived to discharge (pooled prevalence 3%) [8]. With a such dismal prognosis of cardiopulmonary arrest in COVID-19 patients, the rescuers have two options - to flip the patient to supine (while delaying initiation of CPR) or deploy prone CPR.

No difference was found in cardiac arrest outcomes during the COVID-19 pandemic if the code team responded after donning personal protective equipment (PPE) to a patient with in-hospital cardiac arrest [9]. While this may be due to the limited time required to don PPE, turning a patient can take up to 5 minutes and require large healthcare teams, ideally with five or more individuals. This increases the risks associated with cardiac arrest and prolongs the time a patient has no perfusion. Turning a patient requires individuals trained in not only the physical process but also how and when to clamp endotracheal tubes and temporarily cease ventilation, thus creating another barrier [10]. Additionally, cycling hypoxemic patients from prone to supine can aggravate hypoxemia through accidental disconnection of the ventilator and loss of positive end-expiratory pressure [5]. An inadvertent disconnect of the ventilator circuit during position change may also increase the risk of airborne exposure to healthcare personnel. For these reasons, keeping a prone patient in a prone position is beneficial as long as high-quality CPR can be delivered.

Though studies are limited, initial research on this topic has shown that prone CPR can result in the return of spontaneous circulation. A systematic review by Brown et al. evaluated 22 patients who underwent prone CPR and found that 10 individuals, or 45.5%, were rescued by prone CPR [11]. It has been postulated that the more rigid thoracic costovertebral joints should allow more forceful compression, which would create higher pressure in the intra-thoracic venous and arterial conduits and further ventricular compression, thus improving forward flow [12].

In addition to the survival rates, the success of prone CPR is determined by its ability to pump blood throughout the body and its capability to restore physiologic blood pressures. Prone CPR with sternal counter-pressure has been shown to increase intrathoracic pressure, increase systolic blood pressure, and result in near physiologic blood pressures [12]. For patients who have undergone prone CPR, average systolic blood pressure increased to 73 mmHg compared to 48 mmHg in traditional CPR [12]. Mean arterial pressure increased to 46 mmHg in prone CPR compared to 32 mmHg in traditional CPR [12]. A similar recent study comparing prone and supine CPR corroborates these results. It shows that there is a higher mean systolic blood pressure for prone CPR than for supine CPR (79 ±20 vs. 55 ±20 mm Hg), as well as a higher diastolic blood pressure of 17 ±10 and 13 ±7 mmHg [6]. This demonstrates the efficacy of prone CPR as it maintains blood pressure levels high enough to allow oxygenation of vital organs. However, this increase in systolic and mean arterial pressures can increase the risk of future arrest [10]. This risk can be mitigated by using end-tidal capnography and arterial pressure waveforms to assess the risk of cardiovascular arrest and determine whether prone CPR is appropriate [10]. Some cases even show that prone CPR may be superior to traditional CPR as it prevents inferior deviation of the diaphragm [12]. This allows the compressive force in the prone position to be distributed over a smaller area, effectively increasing compression efficiency.

Because studies comparing prone CPR to supinating a patient to perform traditional CPR are limited, we must use our clinical judgment to assess risks vs. benefits. Thus, the American Heart Association recommends that prone patients with an advanced airway in place undergo prone CPR while those without an advanced airway be supinated for traditional CPR [2]. This recommendation prevents the risk of airway collapse in patients undergoing prone CPR who are not intubated and undergoing mechanical ventilation. According to the American Heart Association, the time to initiation of CPR is one of the most critical factors in determining both survival and complication rates of cardiac arrest. The United Kingdom (UK) Resuscitation Council urges current life support guidance to be updated to incorporate patients in the prone position, which includes advocating for anterior-posterior defibrillation [10]. Historical case reports about prone CPR among victims aged six months to 53 years have shown that chest compressions in the prone position were effective when patients were intubated [13].

Prone CPR also reduces the risk of neural damage from position change in patients undergoing neurosurgery who experience a cardiac arrest while their brain or spine is exposed [14].

Limitations of prone CPR

Limited research: Though research in prone CPR has accelerated during the COVID-19 pandemic, it is still a developing area. Further studies are needed to refine the technique, understand its long-term consequences, and evaluate its functionality in real-world conditions. In the systematic review by Anez et al., the literature search located 268 articles; 27 of the 52 included articles were case reports likely due to the rarity of prone CPR and thus may be inclined to publication bias [5]. Most cases of prone CPR reviewed were conducted in the hospital setting on a subset of patients observed by staff who could promptly start resuscitation efforts, thus affecting its comparative efficacy to supine CPR [5]. This difference should be noted when prone CPR is used in real-world and non-ideal situations.

In the cases of prone positioning for ARDS, prone CPR is often trialed on exceptionally sick patients increasing the incidence of a selection bias.

Training of staff: Since knowledge of prone CPR is not as widely disseminated as traditional CPR, healthcare staff may not yet be comfortable performing prone CPR without the proper training [15]. Unlike conventional CPR, there are more requirements to consider before completing prone CPR: patients’ airways must be secured, a hard surface (i.e., sandbags, serum bags) is required, and at least 2-4 appropriately trained staff are needed to perform the procedures (albeit even more individuals may potentially be required to turn the patient supine) [2,5,16]. While the evidence suggests that prone CPR may improve blood flow compared to traditional CPR, prone CPR appears to be more strenuous due to more pressure required to compress stiffer costovertebral joints [12,17]. However, the study by Atkinson et al. also showed that with instructions, adequate CPR was performed by 41% of nurses throughout the cycle, with the number as high as 61% at some stage of the cycle [17]. This was why we used daily briefing with bedside nurses on the procedure of prone CPR during our protocol implementation.

Procedural difficulties: Completing prone CPR may alter or restrict the ability to complete other standard care procedures. Prone CPR may cause the endotracheal/tracheostomy tube to be dislodged [6]. It can also constrain providers from performing a neurological assessment, central venous and arterial access, and/or physical examination [12]. If the patient is prone during surgery, devices such as the Active Compression and Decompression Device (ACDD)-assisted CPR may not be used due to the risk of contamination [18] or due to a lack of knowledge about their appropriate use in this position.

Injuries to patients:** **While there is not much published on medical complications related to prone CPR, it is possible to sustain injuries to the ribs, spine, scapula, clavicles, shoulders, and eyeballs [6]. To better understand the long-term impact and consequences of prone CPR, it is crucial for future studies on prone CPR to assess patients with long-term follow-up.

## Conclusions

Prone CPR offers a perspective that reduces the time to initiation of CPR and can potentially increase survival. Data is available from multiple studies about its utility in clinical situations where the patient is positioned prone and cannot be safely turned supine in a timely fashion. The presence of a definitive airway remains a prerequisite for performing prone CPR due to the risk of airway-related complications without it. It involves a learning curve, and appropriate protocol development and training are required before it is adapted across institutions. We have been able to develop a protocol at our institution with a multi-disciplinary approach toward data analysis, training, and quality control. More research is needed to define both short- and long-term outcomes and complications. Repeated training is going to be required to keep the skills due to the expected low volume of patients that fulfill the criteria for initiation of prone CPR. 
